# Risk Factors of Anterior Circulation Intracranial Aneurysm Rupture: Extracranial Carotid Artery Tortuosity and Aneurysm Morphologic Parameters

**DOI:** 10.3389/fneur.2021.693549

**Published:** 2021-07-12

**Authors:** Yusong Pei, Zhihua Xu, Guobiao Liang, Hai Jin, Yang Duan, Benqiang Yang, Xinxin Qiao, Hongrui You, Dengxiang Xing

**Affiliations:** ^1^Jinzhou Medical University General Hospital of Northern Theater Command Postgraduate Training Base, Shenyang, China; ^2^Department of Radiology, Tongde Hospital of Zhejiang Province, Hangzhou, China; ^3^Department of Neurosurgery, General Hospital of Northern Theater Command, Shenyang, China; ^4^Center for Neuroimaging, General Hospital of Northern Theater Command, Shenyang, China; ^5^Department of Radiology, General Hospital of Northern Theater Command, Shenyang, China; ^6^Center for Medical Data, General Hospital of Northern Theater Command, Shenyang, China

**Keywords:** aneurysm rupture, extracranial carotid artery tortuosity, morphologic parameters, intracranial aneurysm, CTA

## Abstract

**Background:** This study was conducted to explore the risk factors of anterior circulation intracranial aneurysm rupture based on extracranial carotid artery (ECA) tortuosity.

**Methods:** This retrospective study, conducted from January 1, 2017, to March 1, 2021, collected and reviewed the clinical and imaging data of 308 patients with anterior circulation intracranial aneurysm [133 (43.2%) patients in the ruptured aneurysm group; 175 (56.8%) patients in the unruptured aneurysm group]. Computed tomography angiography (CTA) of the head and neck was used to determine the ECA tortuosity (normal, simple tortuosity, kink, coil) and the morphologic parameters of the aneurysms. The relationship of aneurysm rupture to ECA tortuosity and the morphologic parameters were analyzed.

**Results:** After univariate analysis, kink, angle of flow inflow (FA), aspect ratio (AR), aneurysm length (*L*), the distance from the tortuosity to the aneurysm (distance), and size ratio (SR) were significantly correlated with anterior circulation intracranial aneurysm rupture (*p* < 0.05). Spearman correlation analysis showed that ECA tortuosity was correlated with FA and SR (*p* < 0.05). Multiple logistic analyses showed that FA [odds ratio (OR), 1.013; 95% CI, 1.002–1.025], SR (OR, 1.521; 95% CI, 1.054–2.195), and kink (OR, 1.823; 95% CI, 1.074–3.096) were independently associated with aneurysm rupture.

**Conclusion:** Study results suggest that FA, SR, and ECA kink were independent risk factors associated with anterior circulation intracranial aneurysm rupture.

## Introduction

Studies in the literature have reported that intracranial aneurysm prevalence rate can be as high as 3% (1, 2). After aneurysmal rupture, subarachnoid hemorrhage (SAH) often occurs. The incidence varies widely by region, with only 2 per 100,000 cases in China and 22.5 per 100,000 cases in Finland (3). Extracranial carotid artery (ECA) tortuosity is related to connective tissue diseases in which the shape of the ECA is different from the normal shape (4–6). Weibel and Fields (5) first divided ECA into simple tortuosity, kink, and coil according to their degree of tortuosity. ECA tortuosity is often associated with leukoaraiosis, a pathologic appearance of brain white matter, and ischemic stroke, dissecting aneurysms (7–9). However, research on the correlation between ECA tortuosity and intracranial aneurysm was carried out relatively late. It was not until 2017 that Labeyrie et al. (10) conducted a correlation study on ECA tortuosity and the occurrence of intracranial aneurysms. Studies have shown that greater ECA tortuosity will promote hemodynamic changes, leading to the progression of aneurysms (11). Labeyrie et al. (10) and Kliś et al. (12), through subjective observation and quantitative analysis, respectively, both concluded that ECA tortuosity is related to the occurrence of intracranial aneurysms, but it is not a factor for intracranial aneurysm rupture. The reason may be that their studies included the entire intracranial aneurysm, which would bias the experimental results. Taking into account our hypothesis, the ECA may cause rupture of an anterior circulation aneurysm but have little to no effect on posterior circulation aneurysms because of the distance of the ECA from the posterior circulation. Therefore, our study only considers aneurysms in the anterior circulation. The main risk factors for intracranial aneurysm progression and rupture are morphologic parameters and hemodynamics, and the two influence each other (13–15). First, hemodynamics are not easy to measure using computed tomography angiography (CTA). Second, most radiologists use CTA only to measure the morphologic parameters of intracranial aneurysms, and they do not include extracranial parameters. Therefore, we studied the ECA tortuosity and aneurysm morphologic parameters, and we further analyzed what factors were associated with anterior circulation intracranial aneurysm rupture.

## Materials and Methods

### Patients

**Figure 1:**
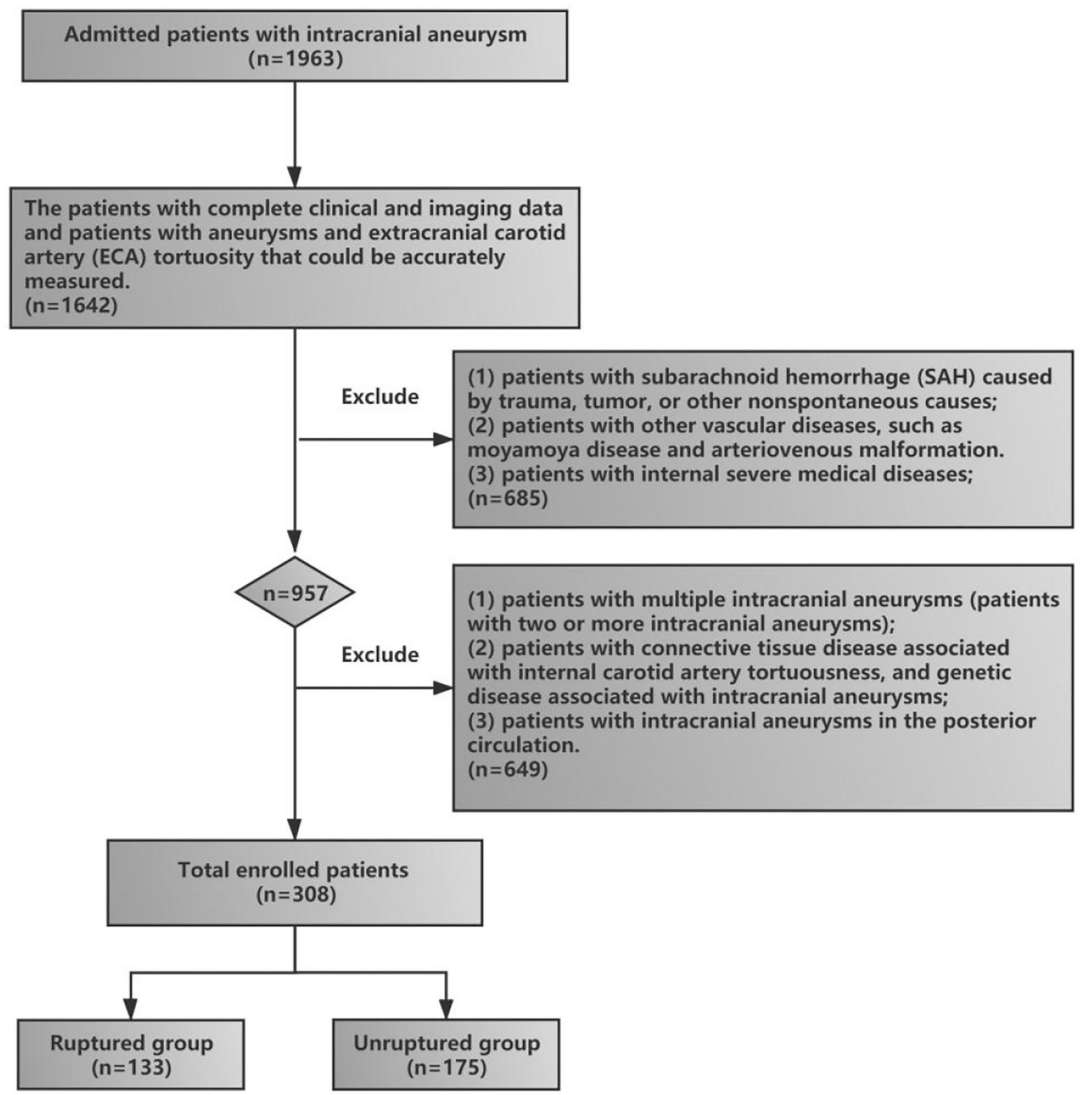
Flowchart of the enrollment of study patients.

This retrospective study, conducted from January 1, 2017, to March 1, 2021, was approved by the Ethics Committee Review Board of The General Hospital of Northern Theater Command, and the patients or their family members provided written informed consent. The clinical and imaging data of patients with anterior circulation intracranial aneurysms were collected and reviewed. The inclusion criteria included (1) patients who were admitted to the hospital for aneurysm rupture and were diagnosed with aneurysm rupture-induced SAH by head and neck CTA (these patients were included in the rupture group), (2) patients with unruptured intracranial aneurysms found on incident and diagnosed by head and neck CTA (these patients were included in the unruptured group), (3) patients with complete clinical and imaging data, and (4) patients with aneurysms and ECA tortuosity that could be accurately measured. The exclusion criteria included (1) patients with SAH caused by trauma, tumor, or other nonspontaneous causes; (2) patients who did not undergo head and neck CTA, or alternatively, the CTA could not distinguish ECA tortuosity; (3) patients with other vascular diseases, such as moyamoya disease and arteriovenous malformation; (4) patients with internal severe medical diseases; (5) patients with multiple intracranial aneurysms (two or more intracranial aneurysms); (6) patients with connective tissue disease associated with internal carotid artery tortuosity and genetic disease associated with intracranial aneurysms (fibromuscular dysplasia, Ehlers–Danlos syndrome, Marfan disease, etc.); and (7) patients with intracranial aneurysms in the posterior circulation. The flowchart of patient enrollment in our study is shown in Figure 1.

### Clinical Information

The sex, age, medications (e.g., aspirin), and risk factors for aneurysms, including current smoking, alcohol use, diabetes mellitus, coronary artery disease, and hypertension, were acquired on admission from all patients.

### Head and Neck CTA Scan and Image Analysis

All patients were scanned using the US GE Discovery CT 750 HD scanner (GE Healthcare, Milwaukee, WI, USA). Patients were placed in the supine position, with their upper limbs lying flat on both sides of their body and the head slightly inclined. The scan range was from the level of the aortic arch to the top of the skull. Scanning parameters were as follows: 100 kV, 120 mAs, 40-mm collimator width, 25-cm field of view, 5-mm layer thickness, 5-mm layer spacing, and iodixanol contrast agent (270 mg/ml) with a dosage of 60 ml and an injection rate of 5 ml per second, *via* elbow vein injection. The original scanned image was processed in thin layers (0.625 mm) by the post-processing workstation to obtain a boneless blood vessel virtual reality (VR) image; two experienced neuroradiologists collected and measured the morphological parameters and ECA tortuosity of the aneurysm and measured them together (Yusong Pei, Xinxin Qiao).

### Determination of Aneurysm Location and Aneurysm Morphology

The criteria for determining aneurysm location (obtained *via* head CTA) were based on the following landmarks: (1) the internal carotid artery, (2) the middle cerebral artery, (3) the anterior cerebral artery, (4) the anterior communicating artery, and (5) the posterior communicating artery. Aneurysm morphology was labeled as regular (no lobes, smooth, no ascus, no protrusion, saccular, or cone).

### Measurement of Intracranial Aneurysm Morphologic Parameters

**Figure 2:**
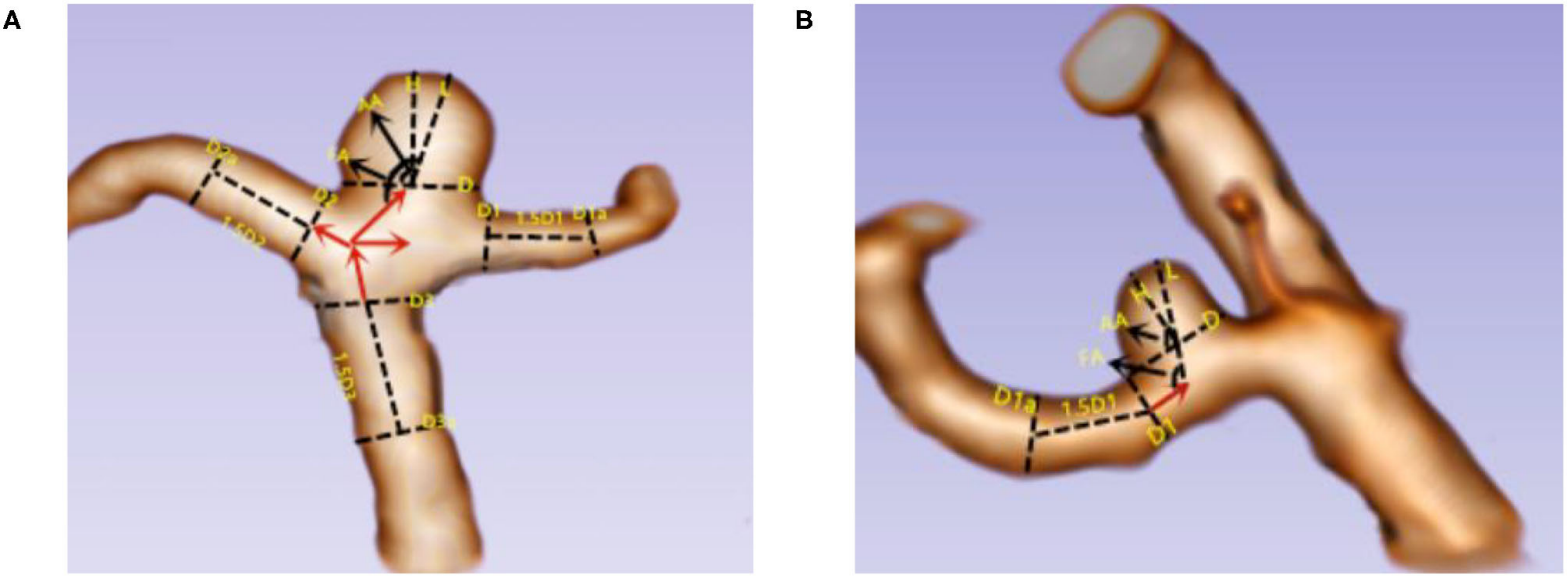
Two kinds of aneurysm parameters were measured. **(A)** End-wall aneurysm; **(B)** side-wall aneurysm. *L* = the maximum height from the diameter of the aneurysm to the wall of the aneurysm; *H* = the maximum vertical distance from the aneurysm diameter plane to the top of the aneurysm; *D* = the width of the two edge lines perpendicular to the aneurysm diameter; Dm, for lateral wall aneurysms, Dm = (D1 + D1a)/2; for triadic aneurysms, Dm = (Dv1 + Dv2 + Dv3)/3, Dv1 = (D1 + D1a)/2, Dv2 = (D2 + D2a)/2, and Dv3 = (D3 + D3a)/2; D1a (D2a, D3a) = along the direction of blood flow, the diameter of the artery at a distance of 1.5 times D1 (D2, D3); FA = the angle between the direction of blood flow and the radial plane of the aneurysm; AA = the angle between the maximum height of the aneurysm and the plane of the aneurysm diameter.

The following list includes the morphologic parameters used in this study and their definitions: (1) aneurysm length (*L*): the maximum height from the diameter of the aneurysm to the wall of the aneurysm; (2) aneurysm height (*H*): the maximum vertical distance from the aneurysm diameter plane to the top of the aneurysm; (3) aneurysm neck diameter (*D*): the width of the two edge lines perpendicular to the aneurysm diameter; (4) the main diameter of the vessel (Dm): for lateral wall aneurysms, Dm = (D1 + D1a)/2; for triadic aneurysms, Dm = (Dv1 + Dv2 + Dv3)/3, Dv1 = (D1 + D1a)/2, Dv2 = (D2 + D2a)/2, and Dv3 = (D3 + D3a)/2, D1a (D2a, D3a) = along the direction of blood flow, the diameter of the artery at a distance of 1.5 times D1 (D2, D3); (5) size ratio (SR): the ratio of the long neck of the aneurysm to the average diameter of the aneurysm-bearing artery; (6) aspect ratio (AR): the ratio of *H* to *D*; (7) angle of flow inflow (FA): the angle between the direction of blood flow and the radial plane of the aneurysm; and 8) aneurysm dip angle (AA): the angle between the maximum height of the aneurysm and the plane of the aneurysm diameter. According to the characteristics of different aneurysms, different measurement methods were used. Aneurysms were divided into end-wall aneurysms and side-wall aneurysms. Aneurysm-related measurement methods are shown in Figure 2.

### Definition of ECA Tortuosity

**Figure 3:**
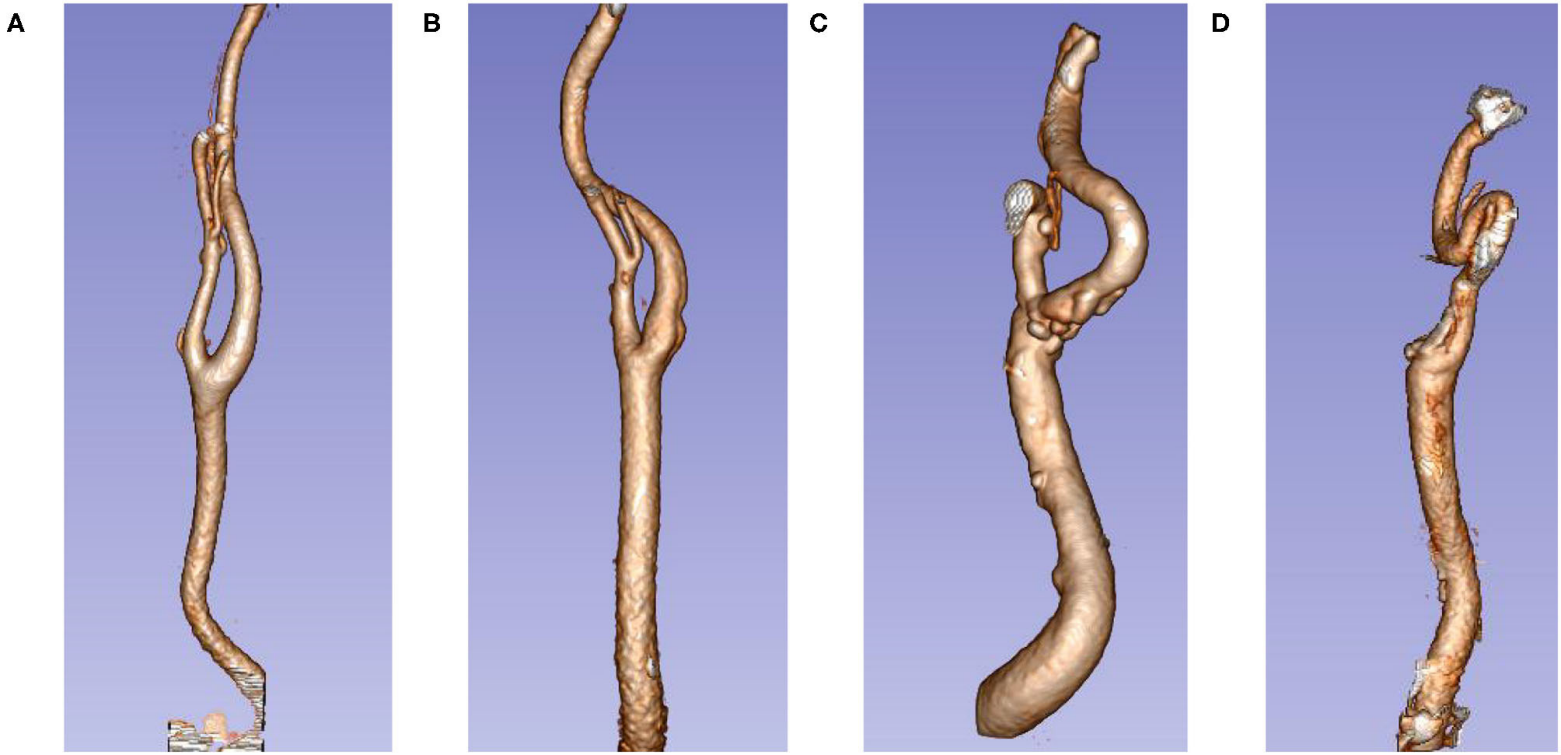
Extracranial carotid artery tortuosity type. **(A)** Normal; **(B)** simple tortuosity; **(C)** kink; **(D)** coil.

According to the standards of Weibel et al. and Paulsen et al. (5, 16), our study divided the ECA tortuosity into four types: (1) normal: ECA is almost linearly shaped; (2) simple tortuosity: ECA becomes slightly tortuous and develops an “S” shape; (3) kink: the ECA becomes more tortuous and develops a “>” or “ < ” shape; and (4) coil: ECA tortuosity is extensive and develops a “U” shape. The distance from the tortuosity to the aneurysm (distance) was also measured. Determination of tortuosity of the internal carotid artery: for an anterior communicating aneurysm, the side with more severe ECA tortuosity on both sides was selected; for other aneurysms, the ECA tortuosity on the side of the ruptured aneurysm was selected (Figure 3).

### Statistical Analysis

Categorical variables were expressed as frequency or percentage. Normally distributed continuous variables were represented by mean and SD, and non-normally distributed data were represented by quartiles and interquartile range (IQR). The independent samples *t*-test was used for normally distributed data, and the Mann–Whitney *U* test was used for non-normally distributed data. Chi-square testing was used for categorical variables. First, univariate analysis of the clinical data and the imaging parameters of the aneurysms in the rupture group and the unruptured group was performed. Second, Spearman correlation was used to analyze the relationship between ECA tortuosity and the morphologic parameters of the intracranial aneurysm. Finally, the factors with significance in the univariate analysis were corrected, and those with *p* <0.10 in the univariate analysis were included in the multivariate logistic analysis. Interobserver agreement was assessed using the kappa statistic (*K*) and single measures statistic (intraclass correlation efficient, ICC). *K* > 0.6 was considered good agreement, whereas *K* > 0.8 was considered excellent; ICC > 0.75 was considered excellent (17–19). All *p*-values were two-sided, and statistical significance was defined as *p* < 0.05. All data were analyzed from December 15, 2020, to January 10, 2021, using the Statistical Package for Social Sciences of Windows, version 24 (IBM Corporation, Armonk, NY, USA).

## Results

### Interreader Agreement in Evaluation of FA, AA, H, D, L, Distance, and ECA Tortuosity

The CTA quality met the diagnostic standard and allowed the neuroradiologist to measure FA, AA, *H, D, L*, distance, and ECA tortuosity; images of some patients were excluded due to poor quality. The agreement between readers was excellent for FA (ICC = 0.976), AA (ICC = 0.962), *H* (ICC = 0.953), *D* (ICC = 0.932), *L* (ICC = 0.974), distance (ICC = 0.968), and ECA tortuosity (*K* = 0.992).

### Univariate Analysis of the Ruptured Group and the Unruptured Group

Of the 308 patients [mean age ± SD, 61 ± 11 years; 144 men (46.7%)] included in the study, the results of the univariate analysis showed that the differences in kink, FA, AR, *L*, distance, and SR were significant (*p* < 0.05) (Table 1).

**Table 1:** Univariate analysis of factors associated with intracranial aneurysm rupture.

	**Ruptured group (*n* = 133)**	**Unruptured group (*n* = 175)**	***p*-value**
Age, years, mean ± SD	59 ± 11	62 ± 11	0.076
**Clinical factors**, ***n*** **(%)**			
Current smoker	51 (38.3%)	64 (36.6%)	0.750
Current drinker	24 (18.0%)	47 (26.9%)	0.069
Hypertension	73 (54.9%)	106 (60.6%)	0.317
Diabetes mellitus	10 (7.5%)	20 (11.4%)	0.252
Coronary disease	6 (4.5%)	15 (8.6%)	0.161
Taking aspirin history	13 (9.8%)	26 (14.9%)	0.184
Sex, M	59 (44.4%)	85 (48.6%)	0.463
**Location**, ***n*** **(%)**			
ICA	35 (26.3%)	61 (34.9%)	0.109
MCA	18 (13.5%)	36 (20.6%)	0.108
ACA	25 (18.8%)	24 (13.7%)	0.227
ACOA	22 (16.5%)	21 (12.0%)	0.255
PCOA	33 (24.8%)	33 (18.9%)	0.207
**ECA tortuosity**, ***n*** **(%)**			
Normal	16 (12.0%)	34 (19.4%)	0.081
Simple tortuosity	54 (40.6%)	88 (50.3%)	0.091
Kink	55 (41.4%)	40 (22.9%)	<0.001
Coil	8 (6.0%)	13 (7.4%)	0.626
Distance	156.3 (133.9–178.7)	160.3 (144.6–186.6)	0.031
**Parameter, IQR**			
FA	110.20 (90.20–125.45)	99.20 (78.80–110.20)	<0.001
AA	70.60 (60.50–97.70)	70.60 (60.0–95.20)	0.519
*H*	2.80 (2.00–3.65)	2.40 (1.80–3.60)	0.151
*D*	2.80 (2.10–3.85)	3.00 (2.40–3.90)	0.130
AR	1.00 (0.74–1.21)	0.92 (0.59–1.12)	0.006
*L*	3.10 (2.60–4.70)	2.80 (2.20–4.10)	0.012
SR	1.96 (1.38–2.26)	1.50 (1.15–2.06)	<0.001
**Morphology**, ***n*** **(%)**			
Ascus	21 (15.8%)	19 (10.9%)	0.202
Irregular	25 (18.8%)	47 (26.9%)	0.098

*SD, standard derivation; IQR, interquartile range; ICA, internal carotid artery; MCA, middle cerebral artery; ACA, anterior cerebral artery; ACOA, anterior communicating artery; PCOA, posterior communicating artery; ECA, extracranial carotid artery; FA, angle of flow inflow; AA, aneurysm dip angle; H, aneurysm height; D, aneurysm neck diameter; AR, aspect ratio; L, aneurysm length; SR, size ratio*.

### Spearman Analysis Between ECA Tortuosity and the Morphologic Parameters of Aneurysms

Results of the Spearman analysis showed that only FA (*r* = 0.312, *p* <0.001) and SR (*r* = 0.210, *p* <0.001) were correlated with ECA tortuosity. The greater the degree of tortuosity of the ECA, the greater the FA and SR (Table 2).

**Table 2:** Spearman analysis of ECA tortuosity and the morphologic parameters of aneurysms.

	**FA**		**SR**	
	***r***	***p***	***r***	***p***
ECA tortuosity	0.312	<0.001	0.210	<0.001

*ECA, extracranial carotid artery; FA, angle of flow inflow; SR, size ratio; r, Spearman correlation coefficient*.

### Multivariate Logistic Analysis of Risk Factors for Aneurysm Rupture

Multivariate logistic analysis takes SAH as the dependent variable and takes age, current drinker, normal, simple tortuosity, kink, distance, FA, AR, *L*, SR, and irregular as independent variables. After multivariate analysis, FA (OR, 1.013; 95% CI, 1.002–1.025; *p* = 0.017), SR (OR, 1.521; 95% CI, 1.054–2.195; *p* = 0.025), and ECA kink (OR, 1.823; 95% CI, 1.074–3.096; *p* = 0.026) were significantly different (*p* <0.05). They were independent risk factors associated with intracranial aneurysm rupture. However, it did not differ in current drinker, age, ECA normal, ECA simple tortuosity, distance, AR, *L*, and irregular (*p* > 0.05) (Table 3).

**Table 3:** Multivariate analysis of anterior circulation intracranial aneurysm rupture risk factors.

	**OR value**	***p*-value**	**95% CI**	
Current drinker	0.578	0.075	0.316	1.056
Age	0.984	0.140	0.962	1.005
Normal	0.911	0.871	0.297	2.799
Simple tortuosity	1.243	0.672	0.454	3.406
Kink	1.823	0.026	1.074	3.096
Distance	0.994	0.120	0.986	1.002
FA	1.013	0.017	1.002	1.025
SR	1.521	0.025	1.054	2.195
AR	1.166	0.566	0.690	1.970
*L*	0.900	0.271	0.745	1.086
Irregular	0.685	0.209	0.380	1.236

*FA, angle of flow inflow; SR, size ratio; AR, aspect ratio; L, aneurysm length*.

## Discussion

Our study found that FA and SR were independently associated with intracranial aneurysm rupture, which is consistent with the studies of Jiang et al. (20) and Juvela (21). Our study also found that ECA kink is an independent risk factor associated with aneurysm rupture. This is inconsistent with the study of Labeyrie et al. (10), whose conclusion was that ECA tortuosity was not associated with rupture. The probable reason for this is that they considered all intracranial aneurysms, whereas we only included intracranial aneurysms from the anterior circulation. Another reason may be that they included a small sample size or a population of people with a different race/ethnicity.

ECA tortuosity can cause the original cranial blood flow pattern to change, which causes abnormal hemodynamics resulting in a change in the vascular structure. Changes in the vascular structure make the aneurysm-bearing artery vulnerable within its environment. Based on the study of the correlation between ECA tortuosity and aneurysm morphologic parameters, we concluded that ECA tortuosity was associated with FA, and Spearman correlation analysis showed that the greater the degree of tortuosity of the ECA, the greater the FA. We suspect that ECA tortuosity causes the blood flow trajectory of an aneurysm-bearing artery to shift, thereby causing its FA to increase. Skodvin et al. (22) pointed out that the larger the FA, the more likely the aneurysm is to rupture. When the FA value increases, which could induce an unfavorable hemodynamic environment, more complicated flow pattern and the wall shear stress (WSS) of the aneurysm can be relatively increased (23), which is likely to cause the aneurysm to rupture (24). Studies have highlighted that FA, as the most common hemodynamics parameter, is the strongest influencing factor leading to aneurysmal rupture (25). However, because the instantaneity of the blood flow of an aneurysm is relatively uncertain, FA serves as an accurate measure at the moment of aneurysm rupture. Therefore, FA has not been regarded as an independent risk factor in clinical practice, and it should be analyzed jointly with other factors. Future studies should perform a combined analysis of ECA kink (as a cofactor) and FA to establish a new system of predicting aneurysmal rupture, to improve the treatment of aneurysms, and ultimately, to prevent rupture.

Our study results suggest that SR was an independent risk factor associated with aneurysmal rupture. SR was first proposed by Dhar et al. (26) to be related to aneurysm rupture. Kang et al. (27) found that the greater the SR value, the higher the risk of aneurysm rupture, which was also found in our study. Compared with the AR value, which only considers the shape of the aneurysm itself, SR introduces the diameter of the aneurysm-bearing artery, which is more comprehensive, and its threshold is more stable (the threshold value of the ratio is ~1.8–2). We concluded that ECA tortuosity was associated with SR, and Spearman correlation analysis showed that the greater the degree of tortuosity of the ECA, the greater the SR. We suspect that ECA tortuosity would first lead to changes in FA, and with long-term changes in FA, it would lead to changes in the SR of intracranial aneurysms.

In recent years, there have been many correlation studies on the influence of morphologic parameters on aneurysm rupture. It is known that AR, SR, *L*, and other related indicators are significantly related to aneurysm rupture, but most of these studies used univariate analyses. Considering the correlation between morphologic parameters, the results of multivariate analyses may deviate greatly from those of univariate analyses. Dhar et al. (26) first conducted a multivariate analysis of the relationship between morphologic parameters and aneurysm rupture and found that only SR and FA were independent risk factors. However, univariate analysis showed that six morphologic factors were significantly correlated with aneurysm rupture. We examined univariate factors, and the results suggest that AR, SR, *L*, kink, and FA were significantly correlated. According to the comparison between the morphologic parameters of ECA and aneurysms, we found that FA and SR were associated with ECA. Before the multivariate logistic analysis, we considered that there might be a colinear relationship between them, but in the end, there was no colinear relationship found. Finally, it was concluded that FA, SR, and ECA kink were independent risk factors associated with aneurysm rupture.

**Figure 4:**
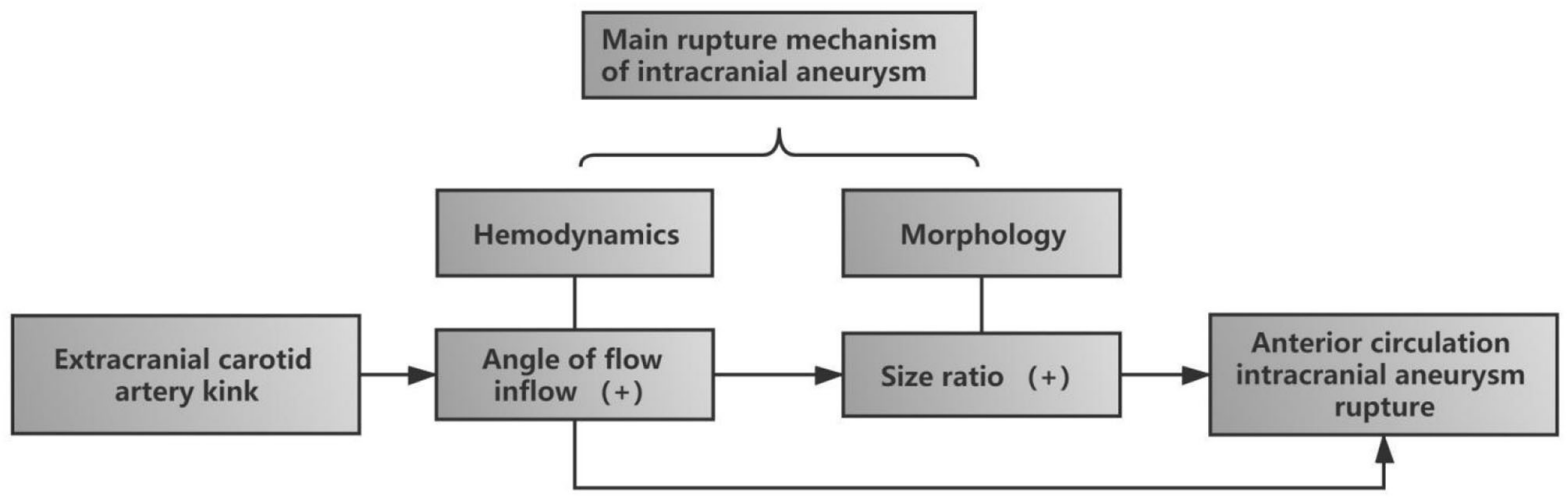
Diagram of the influence of extracranial carotid artery (ECA) kink on the rupture of intracranial aneurysm in anterior circulation. ECA kink first increased the incidence angle of flow inflow, one of the hemodynamics, and with the increase of FA, size ratio, one of the morphological parameters, gradually increased. Finally, the two risk factors together led to the rupture of aneurysm.

In conclusion, our study results suggest that ECA kink was independently associated with the rupture of anterior circulation intracranial aneurysm. The main mechanism of intracranial aneurysm rupture is morphological change and hemodynamic change. Firstly, ECA kink changes hemodynamics, leading to an increase in FA. Secondly, with the increase of FA, the vascular wall was gradually affected, resulting in a gradual change in the morphological parameters (SR) of aneurysms. Finally, both morphological and hemodynamic changes synthetically lead to rupture of intracranial aneurysm (Figure 4).

There are several limitations of our study. First, this study was a retrospective analysis at a single center. For example, considering our hypothesis, if ECA had a coil shape, the risk of intracranial aneurysm rupture was higher, but because of the low incidence of the coil itself and the small sample size (21 cases), our study could not conclude that ECA coil was directly related to rupture. In the future, we will increase the sample size in a multicenter prospective research approach. Second, vasospasm after aneurysm rupture has an effect on the study measurements, but the peak period for vasospasm occurrence is ~5 days. Most of this study was measured within 24 h after rupture; therefore, the effects of vasospasm may have been missed. Third, our study did not include hemodynamics. In the future, we will discuss the influence of hemodynamics, and this study will be more complete. Finally, this research involved the manual measurement of the morphologic parameters of aneurysms, which will inevitably produce certain errors. We will use related software to take more precise measurements in the future.

## Conclusion

Our study results suggest that ECA kink was associated with intracranial anterior circulation aneurysm morphologic parameters. Further, FA, SR, and ECA kink were independent risk factors associated with the rupture of intracranial anterior circulation aneurysms.

## Data Availability Statement

The raw data supporting the conclusions of this article will be made available by the authors, without undue reservation.

## Ethics Statement

The studies involving human participants were reviewed and approved by The Ethics Committee of the General Hospital of the Northern Theater Command of the People's Liberation Army of China. The patients/participants provided their written informed consent to participate in this study.

## Author Contributions

YP, ZX, and YD conceived the project idea and wrote the paper. GL and HJ provided the clinical data and follow-up. XQ and HY collected the imaging data. YP, ZX, and HY provided the imaging analysis. DX sorted medical images and clinical dates. YD and BY supervised the project. All authors contributed to the article and approved the submitted version.

## Conflict of Interest

The authors declare that the research was conducted in the absence of any commercial or financial relationships that could be construed as a potential conflict of interest.

## References

[1] Etminan N Rinkel GJ. Unruptured intracranial aneurysms: development, rupture and preventive management. Nat Rev Neurol. (2017) 13:126. 10.1038/nrneurol.2017.14 28145447

[2] UCAS Japan Investigators Morita A Kirino T Hashi K Aoki N Fukuhara S. The natural course of unruptured cerebral aneurysms in a Japanese cohort. N Engl J Med. (2012) 366:2474–82. 10.1056/NEJMoa1113260 22738097

[3] Ingall T Asplund K Mähönen M Bonita R. A multinational comparison of subarachnoid hemorrhage epidemiology in the WHO MONICA stroke study. Stroke. (2000) 31:1054–61. 10.1161/01.STR.31.5.1054 10797165

[4] Sethi SS Lau JF Godbold J Gustavson S Olin JW. The S curve: a novel morphological finding in the internal carotid artery in patients with fibromuscular dysplasia. Vasc Med. (2014) 19:356–62. 10.1177/1358863X14547122 25135311

[5] Weibel J Fields WS. Tortuosity, coiling, and kinging of the internal carotid artery. Neurology. (1965) 15:7–18. 10.1212/WNL.15.1.7 14257832

[6] Morris SA Orbach DB Geva T Singh MN Gauvreau K Lacro RV. Increased vertebral artery tortuosity index is associated with adverse outcomes in children and young adults with connective tissue disorders. Circulation. (2011) 124:388–96. 10.1161/CIRCULATIONAHA.110.990549 21730308

[7] Yu K Zhong T Li L Wang J Chen Y Zhou H. Significant association between carotid artery kinking and leukoaraiosis in middle-aged and elderly Chinese patients. J Stroke Cerebrovasc Dis. (2015) 24:1025–31. 10.1016/j.jstrokecerebrovasdis.2014.12.030 25817620

[8] Pancera P Ribul M Presciuttini B Lechi A. Prevalence of carotid artery kinking in 590 consecutive subjects evaluated by Echocolordoppler. Is there a correlation with arterial hypertension? J Intern Med. (2000) 248:7–12. 10.1046/j.1365-2796.2000.00611.x 10947875

[9] Kim BJ Yang E Kim NY Kim MJ Kang DW Kwon SU. Vascular tortuosity may be associated with cervical artery dissection. Stroke. (2016) 47:2548–52. 10.1161/STROKEAHA.116.013736 27531344

[10] Labeyrie PE Braud F Gakuba C Gaberel T Orset C Goulay R. Cervical artery tortuosity is associated with intracranial aneurysm. Int J Stroke. (2017) 12:549–52. 10.1177/1747493016687577 28073311

[11] Riccardello GJ Jr Shastri DN Changa AR Thomas KG Roman M Prestigiacomo CJ. Influence of relative residence time on side-wall aneurysm inception. Neurosurgery. (2018) 83:574–81. 10.1093/neuros/nyx433 28945849

[12] Kliś KM Krzyzewski RM Kwinta BM Stachura K Gasowski J. Tortuosity of the internal carotid artery and its clinical significance in the development of aneurysms. J Clin Med. (2019) 8:237. 10.3390/jcm8020237 30759737 PMC6406528

[13] Murayama Y Takao H Ishibashi T Saguchi T Ebara M Yuki I. Risk analysis of unruptured intracranial aneurysms: prospective 10-year cohort study. Stroke. (2016) 47:365–71. 10.1161/STROKEAHA.115.010698 26742803

[14] Cebral JR Mut F Weir J Putman CM. Association of hemodynamic characteristics and cerebral aneurysm rupture. AJNR Am J Neuroradiol. (2011) 32:264–70. 10.3174/ajnr.A2274 21051508 PMC3070915

[15] Schnell S Ansari SA Vakil P Wasielewski M Carr ML Hurley MC. Three-dimensional hemodynamics in intracranial aneurysms: influence of size and morphology. J Magn Reson Imaging. (2014) 39:120–31. 10.1002/jmri.24110 24151067 PMC3865211

[16] Paulsen F Tillmann B Christofides C Richter W Koebke J. Curving and looping of the internal carotid artery in relation to the pharynx: frequency, embryology and clinical implications. J Anat. (2000) 197:373–81. 10.1046/j.1469-7580.2000.19730373.x 11117624 PMC1468139

[17] Mcgraw KO Wong SP. Forming inferences about some intraclass correlation coefficients. Psychol Methods. (1996) 1:390. 10.1037/1082-989X.1.1.30

[18] Shrout PE Fleiss JL. Intraclass correlations: uses in assessing rater reliability. Psychol Bull. (1979) 86:420. 10.1037/0033-2909.86.2.420 18839484

[19] Landis JR Koch GG. The measurement of observer agreement for categorical data. Biometrics. (1977) 33:159–74. 10.2307/2529310 843571

[20] Jiang P Liu Q Wu J Chen X Li M Li Z. A novel scoring system for rupture risk stratification of intracranial aneurysms: a hemodynamic and morphological study. Front Neurosci. (2018) 12:596. 10.3389/fnins.2018.00596 30233292 PMC6133991

[21] Juvela S. Scoring of growth of unruptured intracranial aneurysms. J Clin Med. (2020) 9:3339. 10.3390/jcm9103339 33080974 PMC7603243

[22] Skodvin TØ Evju Ø Sorteberg A Isaksen JG. Prerupture intracranial aneurysm morphology in predicting risk of rupture: a matched case-control study. Neurosurgery. (2019) 84:132–40. 10.1093/neuros/nyy010 29529238

[23] Mo X Meng Q Yang X Li H. The impact of inflow angle on aneurysm hemodynamics: a simulation study based on patient-specific intracranial aneurysm models. Front Neurol. (2020) 11:534096. 10.3389/fneur.2020.534096 33424734 PMC7785798

[24] Shi Z Chen G Z Mao L Li X L Zhou C S. Machine learning-based prediction of small intracranial aneurysm rupture status using CTA-derived hemodynamics: a multicenter study. AJNR Am J Neuroradiol. (2021) 42:648–54. 10.3174/ajnr.A7034 33664115 PMC8041003

[25] Lin N Ho A Gross BA Pieper S Frerichs KU Day AL. Differences in simple morphological variables in ruptured and unruptured middle cerebral artery aneurysms. J Neurosurg. (2012) 117:913–9. 10.3171/2012.7.JNS111766 22957531

[26] Dhar S Tremmel M Mocco J Kim M Yamamoto J Siddiqui AH. Morphology parameters for intracranial aneurysm rupture risk assessment. Neurosurgery. (2008) 63:185–96. 10.1227/01.NEU.0000316847.64140.81 18797347 PMC2570753

[27] Kang H Ji W Qian Z Li Y Jiang C Wu Z. Aneurysm characteristics associated with the rupture risk of intracranial aneurysms: a self-controlled study. PLoS ONE. (2015) 10:e0142330. 10.1371/journal.pone.0142330 26540158 PMC4634979

